# A comparison of emergency airway management between neuromuscular blockades alone and rapid sequence intubation: an analysis of multicenter prospective study

**DOI:** 10.1186/s13104-016-2338-2

**Published:** 2017-01-03

**Authors:** Nobuhiro Sato, Yusuke Hagiwara, Hiroko Watase, Kohei Hasegawa, on behalf of the Japanese Emergency Medicine Network investigators

**Affiliations:** 1 Department of Emergency and Critical Care Medicine, Niigata City General Hospital 463-7, Shumoku, Chuo-ku, Niigata, 950-1197 Japan; 2 Department of Pediatric Emergency and Critical Care Medicine, Tokyo Metropolitan Children’s Medical Center, Fuchu, Tokyo, Japan; 3 Department of Radiology, University of Washington, Seattle, WA USA; 4 Departments of Emergency Medicine, Massachusetts General Hospital, Harvard Medical School, Boston, MA USA

**Keywords:** Neuromuscular blockades, Airway, Intubation, Emergency department

## Abstract

**Background:**

Although airway management with neuromuscular blockade (NMB) alone is discouraged in the emergency department (ED), our previous study demonstrated that many patients were intubated using NMBs alone without sedatives. To refute this practice, we sought to compare the intubation success and adverse event rates between NMBs only and rapid sequence intubation (RSI).

**Methods:**

This is a secondary analysis of the data from a prospective observational study of ED patients in 13 hospitals who underwent emergency airway management from April 2010 to August 2012. The primary outcome was intubation success rate on first attempt. The secondary outcomes were the intubation success rate in ≤2 attempts and the intubation-related adverse event rate. We compared these outcomes between intubation attempts using NMB alone and RSI. We fit multivariable logistic regression models adjusting for potential confounders (age, sex, weight, primary indication for intubation, and training level of intubators).

**Results:**

Overall, 852 patients were eligible for this analysis, with 114 (13%) intubated with NMB alone and 738 (87%) with RSI. Between the NMB-alone and RSI groups, no significant differences were observed in the success rate on the first attempt (70 vs. 73%; P = 0.48) or in ≤2 attempts (89 vs. 91%; P = 0.46), or in the adverse event rate (11 vs. 12%; P = 0.58). Similarly, after adjusting for confounders, no significant differences were observed in any of these outcomes (all P > 0.05).

**Conclusions:**

In this analysis of data from a large multicenter study of ED patients, we found no superior effectiveness of intubation with NMB alone when compared to RSI. Our data lend significant support to the concept that intubation with NMB alone should be avoided in the ED.

## Background

Emergency airway management is a critical intervention conducted in emergency departments (EDs). Higher intubation success rates have been demonstrated when rapid sequence intubation (RSI)—simultaneous administration of sedatives and neuromuscular blockade (NMB)—is deployed. Although the use of sedatives might cause adverse events such as hypotension depending on the dosage and the administered drug (e.g. thiopental or propofol) [1–3], RSI is generally recommended as the first-line method in emergency airway management [4, 5]. Yet, our previous multicenter study in Japan demonstrated that a substantial number of ED patients underwent airway management using only NMBs without sedatives [6]. As NMB lacks the effects of sedation, amnesia, and analgesia, this approach results in an unsettling and uncomfortable experience in non-comatose patients [7, 8]. Therefore, emergency airway management with NMB alone should be avoided. However, to our knowledge, no studies have examined the effectiveness of emergency airway management with NMB alone to refute this approach.

In this context, by using the data from a multicenter prospective study of ED airway management, we aimed to compare the intubation success and adverse event rates between the patients intubated with NMB alone and those intubated with RSI.

## Methods

### Study design, setting, and participants

This study was a secondary analysis of the Japanese Emergency Airway Network (JEAN) Registry, a prospective observational multicenter data registry designed to characterize the current ED airway management in Japan. The study setting, methods of measurement, and measured variables were as previously described [6, 9–11]. Briefly, JEAN was initiated in April 2010 as consortium of 13 academic and community medical centers across Japan. All 13 EDs had emergency attending physicians, and 12 had affiliations with an emergency medicine residency program. The participating institutions were certified as Level I (n = 11) or Level II equivalent (n = 2) trauma centers and had a mean ED census of approximately 29,000 patient visits per year (range 10,000–67,000). In this multicenter observational study with an aim to describe the current airway management in Japanese EDs, we did not specify the drugs or dosages to be used. A prefixed or predetermined dose was not used across the sites. The Institutional Review Board of each participating hospital (Fukui University Hospital, Fukui Prefectural Hospital, Japanese Red Cross Medical Center of Wakayama, Kameda Medical Center, National Center for Global Health and Medicine, Nagoya Ekisaikai Hospital, Obama Municipal Hospital, Okinawa Chubu Prefectural Hospital, Osaka Saiseikai Senri Hospital, Shonan Kamakura General Hospital, Kurashiki Central Hospital, St. Marianna University School of Medicine Hospital and Niigata City General Hospital) approved the protocol with a waiver of informed consent obtained prior to data collection because it was not feasible to obtain consent from patients who require a life-saving emergency procedure-emergency airway management, and this observational study was considered a “minimal risk” study.

Data were gathered prospectively over a 29-month period (April 2010 to August 2012). Adult and pediatric patients who presented to one of these EDs and underwent emergency intubation were eligible for inclusion.

### Outcome measures

The primary outcome measure was intubation success on first attempt. The secondary outcome measures were intubation success in ≤2 attempts and intubation-related adverse event rate. Intubation success was defined as proper placement of the endotracheal tube through the vocal cords with confirmation by quantitative or colorimetric end-tidal carbon dioxide monitoring [4, 6, 9, 10]. We defined the intubation-related adverse events as any adverse events that were potentially related to the procedure itself or occurred during the ED course after intubation. We did not define a specific time-period of intubation-related adverse events, as half-life of sedatives and neuromuscular blocking agents and the physiologic reserve of patients varied widely. Adverse event included esophageal intubation, main bronchial intubation, lip or dental trauma, vomiting, airway trauma, dysrhythmia, hypotension, hypoxemia and death [9]. Esophageal intubation was defined as misplacement of the tracheal tube in the upper esophagus or hypopharynx with a lapse of time and desaturation (pulse oximetry saturation <90%) before the removal of the misplaced tube. Vomiting was defined as gastric contents that required suction removal during laryngoscopy in a previously clear airway. A previous clear airway was defined as airway without visualized gastric contents during laryngoscopy. Hypoxemia was defined as pulse oximetry saturation <90% during an intubation attempt that was potentially-related to the intubation procedure, not secondary to esophageal intubation. Therefore, unchanged hypoxemia was not considered as an adverse event. Hypotension was defined as systolic blood pressure <90 mm Hg. Cardiac arrest included asystole or pulseless electric activity and cardiopulmonary resuscitation during or after intubation. By contrast, we excluded patients who already had cardiac arrest before the intubation procedures.

### Data analysis

For the purpose of this analysis, we identified patients who underwent their first intubation attempt using NMB alone and those with RSI. We excluded patients involving cardiopulmonary arrest prior to emergency airway management, subsequent intubation with alternate methods when the first attempt failed. We also excluded intubation using non-direct-laryngoscopy (e.g. video laryngoscopy or fibroscopy) or the use of a gum-elastic-bougie because all cases which used these devices were intubated with only RSI.

We compared the outcomes between intubation attempts using NMB alone and RSI, by fitting two logistic regression models (unadjusted and adjusted for selected variables) using each of the three outcomes as dependent variables. A set of potential confounders was selected a priori based on biological plausibility and a priori knowledge. These included age, sex, body weight, primary indication for intubation (shock, altered mental status, or others) [2, 12, 13], and training level of intubator [4, 9, 10, 14, 15]. In the sensitivity analyses, we stratified the model by indication and intubator characteristics. All analyses were conducted with JMP statistical software version 9 (SAS Institute Inc., Cary, NC, USA).

## Results

**Fig. 1:**
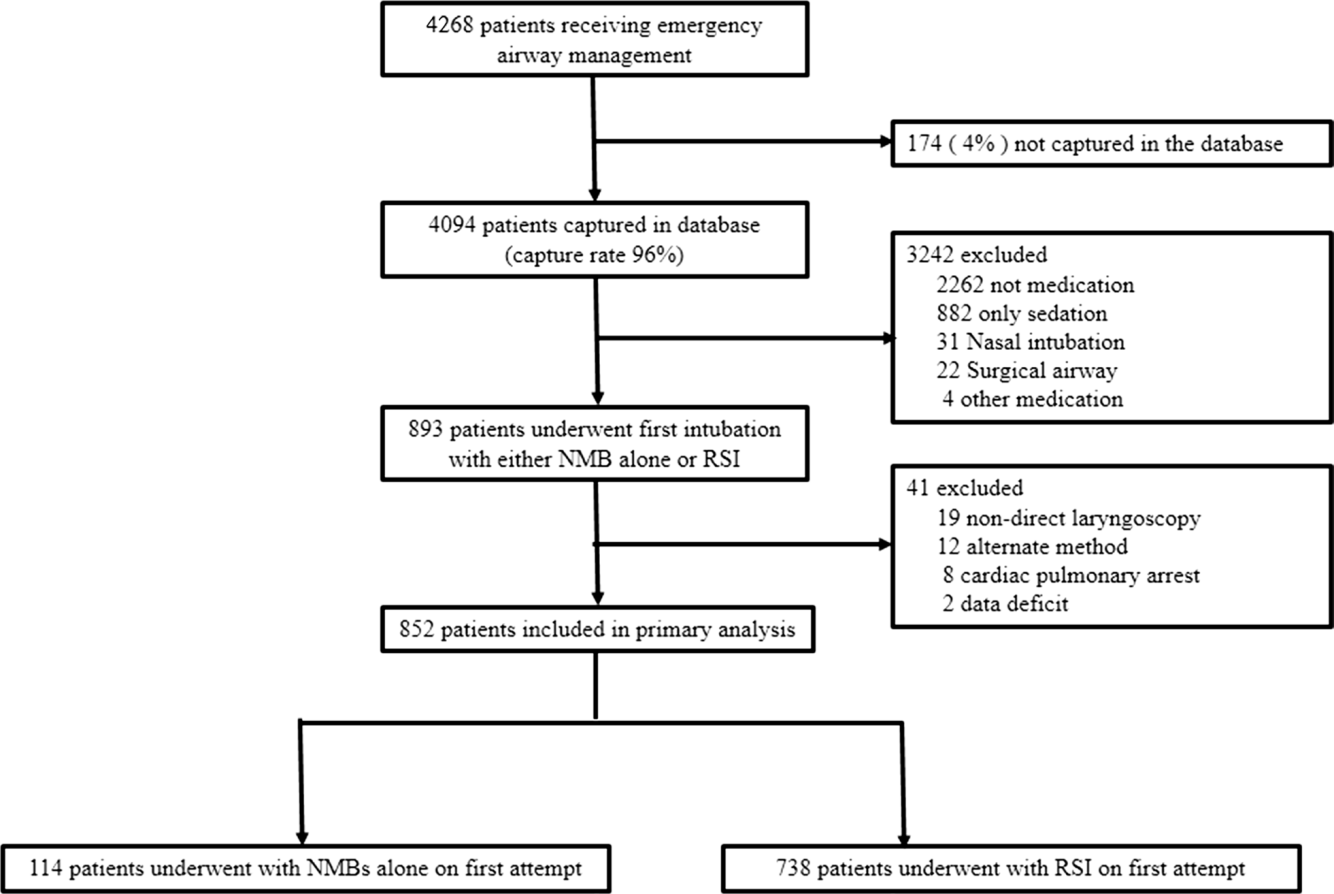
Study flow

During the 29-month period, 4094 encounters (capture rate, 96%) were recorded in the registry. Of these, 893 patients underwent the first intubation attempt using either NMB alone or RSI. Excluded patients were those who were intubated with non-direct-laryngoscopy (n = 19), underwent subsequent intubation attempts with alternate methods (n = 12), had cardiopulmonary arrest (n = 8), and had missing data for an outcome (n = 2). The remaining 852 patients were eligible for analyses (Fig. 1).

Of the patients analyzed, 114 (13%) underwent the first intubation attempt with NMB alone and 738 (87%) with RSI. At the *ED*-level, the proportion of intubations with NMB alone ranged from 0 to 24% (median, 5%; interquartile range [IQR], 0–13%). Patient characteristics for the two groups are shown in Table 1. Overall, the median age was 63 years, and 97% were adults. Compared to the RSI group, patients who underwent emergency airway management with NMB alone were younger, and more likely to have altered mental status and to be intubated by resident physicians (all P < 0.05).

**Table 1:** Characteristics of patients receiving airway management in the emergency department

Patient characteristic	All (n = 852)	NMB alone (n = 114)	RSI (n = 738)	P value
Age, median (IQR), years	63 (48–76)	57 (39–72)	64 (50–76)	0.001
Age ≥18 years, n (%)	829 (97)	112 (98)	717 (97)	0.50
Female sex, n (%)	327 (38)	42 (37)	285 (39)	0.72
Body weight, mean (SD), kg	60 (14)	62 (13)	59 (14)	0.07
Primary indication, n (%)^a^				<0.0001
Medical encounters	668 (78)	96 (84)	572 (78)	
Altered mental status	301 (35)	78 (68)	223 (30)	
Respiratory failure	241 (28)	5 (4)	236 (32)	
Shock	104 (12)	12 (11)	89 (12)	
Airway obstruction	9 (1)	0 (0)	10 (1)	
Asthma	6 (1)	0 (0)	6 (1)	
Other medical	7 (1)	0 (0)	7 (1)	
Trauma encounter	184 (22)	18 (16)	166 (22)	
Head trauma	74 (9)	12 (11)	62 (8)	
Shock	59 (7)	5 (4)	54 (7)	
Multiple trauma	8 (1)	0 (0)	8 (1)	
Facial/neck trauma	15 (2)	1 (1)	14 (2)	
Burn/inhalation	22 (3)	0 (0)	22 (3)	
Other trauma	6 (1)	0 (0)	6 (1)	
Type of sedatives, n (%)^a^				
Midazolam	420 (49)	0 (0)	420 (57)	
Diazepam	100 (12)	0 (0)	100 (14)	
Propofol	98 (12)	0 (0)	98 (13)	
Ketamine	83 (10)	0 (0)	83 (11)	
Opioid	17 (2)	0 (0)	17 (2)	
Others	20 (2)	0 (0)	20 (3)	
Type of NMB, n (%)^a^				
Rocuronium	601 (71)	81 (71)	520 (70)	
Vecuronium	180 (21)	27 (24)	153 (21)	
Succinylcholine	71 (8)	6 (5)	65 (9)	
Specialty of first intubator, n (%)^a^				0.016
Emergency physician	192 (23)	21 (18)	171 (23)	
Emergency medicine resident	157 (18)	24 (21)	133 (18)	
Transitional year resident^b^	381 (45)	62 (54)	319 (43)	
Other	122 (14)	7 (6)	115 (16)	

*IQR* interquartile range, *SD* standard deviation, *NMB* neuromuscular blockade *RSI* rapid sequence intubation

^a^Percentages may not equal 100 due to rounding

^b^Defined as post graduate years 1 or 2

Between the NMB-alone and RSI groups, no significant differences were observed in the success rate on the first attempt (70 vs. 73%; unadjusted odds ratio [OR], 0.9; 95% confidence interval [CI] 0.6–1.3; P = 0.48; Table 2) or in ≤2 attempts (89 vs. 91%; unadjusted OR, 0.8; 95% CI 0.4–1.5; P = 0.46), or in the adverse event rate (11 vs. 12%; unadjusted OR, 0.8; 95% CI 0.4–1.6; P = 0.58). Similarly, after adjusting for confounding factors, no significant differences were observed in the success rate on the first attempt (adjusted OR, 1.1; 95% CI 0.7–1.7; Table 3) or in ≤2 attempts (adjusted OR, 1.2; 95% CI 0.6–2.3), or in the adverse event rate (adjusted OR, 1.2; 95% CI 0.6–2.4). In the sensitivity analyses, the non-significant results persisted with stratification by indication and intubator characteristics (Table 4).

**Table 2:** Unadjusted success rates and adverse event rates, according to intubation method

	n (%)			Unadjusted OR for NMB alone	P value
	All (n = 852)	NMB alone (n = 114)	RSI (n = 738)	OR (95% CI)	
Successful on 1st attempt	621 (73)	80 (70)	541 (73)	0.9 (0.6–1.3)	0.48
Successful in ≤2 attempts	771 (90)	101 (89)	670 (91)	0.8 (0.4–1.5)	0.46
Adverse events^a^	103 (12)	12 (11)	91 (12)	0.8 (0.4–1.6)	0.58

*NMB* neuromuscular blockade, *RSI* rapid sequence intubation, *OR* odds ratio, *CI* confidence interval

^a^Patients may have more than 1 adverse event

**Table 3:** Multivariable associations of airway management methods with success rates and adverse event rates

	Successful on 1st attempt	Successful in ≤2 attempts	Adverse events
	Adjusted OR	Adjusted OR	Adjusted OR
	(95% CI)	(95% CI)	(95% CI)
Primary exposure			
NMB alone	1.1 (0.7–1.7)	1.2 (0.6–2.3)	1.2 (0.6–2.4)
RSI	1 (reference)	1 (reference)	1 (reference)
Covariate			
Age	1.0 (1.0–1.0)	1.0 (1.0–1.0)	1.0 (1.0–1.0)
Female sex	1.0 (0.7–1.3)	1.1 (0.6–1.8)	0.6 (0.4–1.0)
Body weight	1.0 (1.0–1.0)	1.0 (1.0–1.0)	1.0 (1.0–1.0)
Primary indication			
Shock	1.3 (0.8–2.1)	1.7 (0.8–3.9)	0.5 (0.2–1.0)
Altered mental status	1.2 (0.8–1.8)	1.3 (0.8–2.3)	0.9 (0.6–1.5)
Others	1 (reference)	1 (reference)	1 (reference)
Intubator			
Emergency physician	1 (reference)	1 (reference)	1 (reference)
Emergency medicine resident	0.4 (0.2–0.8)	0.4 (0.1–1.3)	1.5 (0.7–3.2)
Traditional year resident^a^	0.1 (0.1–0.2)	0.1 (0.03–0.3)	2.0 (1.1–3.8)
Other	0.3 (0.2–0.6)	0.4 (0.1–1.3)	2.1 (1.0–4.4)

*NMB* neuromuscular blockade, *RSI* rapid sequence intubation, *OR*, odds ratio, *CI* confidence interval

^a^Defined as post graduate years 1 or 2

**Table 4:** Multivariable associations of airway management methods with success rates and adverse event rates

Stratification	Successful on 1st attempt	Successful in ≤2 attempts	Adverse events
	Adjusted OR	Adjusted OR	Adjusted OR
	(95% CI)	(95% CI)	(95% CI)
Indication			
Medical encounters	1.2 (0.7–2.1)	1.4 (0.6–2.9)	1.2 (0.6–2.7)
Trauma encounters	0.5 (0.1–1.8)	0.5 (0.02–2.8)	0.8 (0.2–5.8)
Intubator characteristics			
Emergency physician	1.3 (0.5–3.1)	Not analysed^a^	1.4 (0.4–6.2)
Non-emergency physician	1.0 (0.6–1.7)	1.4 (0.6–2.8)	1.1 (0.5–2.5)

*NMB* neuromuscular blockade, *RSI* rapid sequence intubation, *OR* odds ratio, *CI* confidence interval

^a^All intubations successful within 2nd attempt

## Discussion

In our analysis of a large multicenter study of patients who underwent intubation in the ED, we observed that many patients underwent intubation attempts with NMB alone, with wide inter-hospital variations in the use of this method. Our data also demonstrated no significant differences in rates of intubation success or adverse events between the NMB-alone and RSI groups.

### Results in context

To our knowledge, the use of NMB-alone intubation in the ED has not been addressed in the literature, making our present findings difficult to compare. However, previous studies have reported that absence of concurrent sedation was common among patients receiving long-acting neuromuscular paralysis during transportation between hospitals and NMB-alone or inadequate sedation in non-comatose patients was an avoidable medical error [16–18]. One may argue that emergency airway management with NMB alone has advantages over that with RSI—e.g. a lower frequency of hypotension [3]. However, our data did not demonstrate superior effectiveness of intubation with NMB alone in either the adverse event rate or the success rate. This result persisted with several statistical assumptions. Additionally, paralysis without sedation causes pain and psychogenic trauma as well as sympathetic autonomic discharge leading to hypertension and tachycardia, which might worsen intracranial hemorrhage, vascular dissection, and other conditions [7, 8]. As the use of etomidate has been discouraged in Japan due to its spectrum of adverse effects, which have been addressed in previous studies, ketamine is a potential alternative to induce sedation in hemodynamically unstable patients [3, 19–22]. Collectively, our findings lend significant support to the concept that airway management with NMB alone should be avoided in the ED.

### Variations in emergency airway management across EDs

We are struck by a high degree of variation in the use of NMB alone across the participating EDs. The reasons are likely multifocal. Potential explanations include differences in patient populations, physician preferences, procedural experience levels, training background, and institutional policies. In contrast, our previous study indicated a lack of robust evidence-based airway management education and a peer-review process for residency training programs in Japan [6]. Any or a combination of these factors might explain, at least in part, the wide inter-hospital variation in intubation practice observed in this study.

### Limitations

Our study has several potential limitations. First, passive surveillance introduces the potential for self-reporting bias. Underestimation of adverse event rates is therefore possible. However, we used a standardized system with structured data forms, uniform definitions, and a high capture rate [4]. Additionally, assuming a similar underestimation rate of the outcome between groups, this non-differential misclassification would not have biased our inference. Second, our data predominantly consists of academic EDs in Japan. Our inferences might therefore not be generalizable to other clinical settings. Finally, our inferences might be confounded by unmeasured factors, such as levels of consciousness, pre-intubation blood pressure and the procedural experience of the operators. Granular data on the degree of altered mental status (e.g. Glasgow Coma Scale) would be informative. However, in the JEAN study, data on this medical condition were not collected. We adjusted for the primary indication and training level of the intubator. Although a randomized trial would help determine the efficacy of airway management with RSI compared to NMB alone, such a trial would be unethical. As an alternative, our prospective observational data reflect the effectiveness of airway management methods in the natural setting of a “real” population.

## Conclusions

In this multicenter study of ED airway management, we found that many patients underwent intubation attempts with NMBs without sedatives and that there was a marked inter-hospital variation in the use of this approach. We also found no significant differences in the success or adverse event rates between intubation with NMB alone and that with RSI. Our data lend significant support to the concept that emergency airway management with NMB alone should be avoided. Our findings should encourage healthcare providers and policy makers to improve the quality of ED airway management and decrease these inter-hospital variations.

## Abbreviations

NMB: neuromuscular blockade
ED: emergency department
RSI: rapid sequence intubation
JEAN: Japanese Emergency Airway Network
IQR: interquartile range
OR: odds ratio
CI: confidence interval
